# Rapid preparation of rare ginsenosides by acid transformation and their structure-activity relationships against cancer cells

**DOI:** 10.1038/srep08598

**Published:** 2015-02-26

**Authors:** Kai Quan, Qun Liu, Jin-Yi Wan, Yi-Jing Zhao, Ru-Zhou Guo, Raphael N. Alolga, Ping Li, Lian-Wen Qi

**Affiliations:** 1 State Key Laboratory of Natural Medicines, China Pharmaceutical University, No. 24 Tongjia Lane, Nanjing 210009, China

## Abstract

The anticancer activities of ginsenosides are widely reported. The structure-activity relationship of ginsenosides against cancer is not well elucidated because of the unavailability of these compounds. In this work, we developed a transformation method to rapidly produce rare dehydroxylated ginsenosides by acid treatment. The optimized temperature, time course, and concentration of formic acid were 120°C, 4 h and 0.01%, respectively. From 100 mg of Rh1, 8.3 mg of Rk3 and 18.7 mg of Rh4 can be produced by acid transformation. Similarly, from 100 mg of Rg3, 7.4 mg of Rk1 and 15.1 mg of Rg5 can be produced. From 100 mg of Rh2, 8.3 mg of Rk2 and 12.7 mg of Rh3 can be generated. Next, the structure-activity relationships of 23 ginsenosides were investigated by comparing their cytotoxic effects on six human cancer cells, including HCT-116, HepG2, MCF-7, Hela, PANC-1, and A549. The results showed that: (1) the cytotoxic effect of ginsenosides is inversely related to the sugar numbers; (2) sugar linkages rank as C-3 > C-6 > C-20; (3) the protopanaxadiol-type has higher activities; (4) having the double bond at the terminal C20-21 exhibits stronger activity than that at C20-22; and (5) 20(*S*)-ginsenosides show stronger effects than their 20(*R*)-stereoisomers.

Ginseng is one of the best-selling herbal products in the world. It has gained increasing attention in the treatment of cardiovascular diseases, diabetes and central nervous diseases^1^,^2^,^3^. It is generally believed that triterpenoid saponins called ginsenosides are the major active constituents, and these are also used as the quality control markers^4^,^5^. There is also a popular use of ginsenosides with prominent efforts on anticancer activities associated with cell proliferation^6^, apoptosis^7^, angiogenesis^8^, oxidative stress^9^, inflammation^10^ and metastasis of cancer^11^.

Most ginsenosides share a dammarane triterpenoid structure. Differences in sugar types, numbers and attachment positions provide diversity in structures. By the presence of a hydroxyl moiety at C-6, ginsenosides can be divided into two groups, the protopanaxadiol (PPD) and protopanaxatriol (PPT) group. Ginsenoside Rb1, Rd, Rc, and Rb2 are the major compounds of the PPD-type (Fig. 1a) present in ginseng herbs, with sugar moieties attached to the *β*-OH at C-3 and/or C-20. Ginsenosides Re, Rg1, Rg2, Rf, and Rh1 are the main compounds of the PPT-type (Fig. 1b), with sugar moieties linked to the *α*-OH at C-6 and/or *β*-OH at C-20.

Besides naturally occurring substances, some rare ginsenosides with less sugar moieties and hydroxyl groups continue to be identified. Since most of rare ginsenosides have been commercially unavailable, various methods were developed to generate them, such as chemical treatment^12^, heating^13^, enzymatic^14^ and microbial^15^,^16^ treatment. Wang et al. observed that transformation of ginsenosides by artificial gastric juice showed rapid degradation of ginsenosides and could increase cytotoxicity toward cancer cells^17^. As the main functional component detected in blood or organs after oral administration of ginsenosides, large quantities of compound K (CK) can be produced from cheap monosaccharide via microbial fermentation^18^. We have previously reported that a steaming process caused extensive conversion of ginsenosides in *Panax ginseng* to new degraded ones^13^. The major markers including Rh3, Rk3, Rh4, Rk1, Rk2 and Rg5 (Fig. 1c) were observed in the steamed ginseng, but not detected in raw samples. Rare ginsenosides were reported to show remarkable chemopreventive effects. For example, ginsenoside Rk1 could induce both G1 phase arrest and autophagy at an earlier stage of treatment in HepG2 cells. Rk1 could inhibit telomerase activity and induce apoptosis in HepG2 cells^19^,^20^.

To date, efficient methods are limited for producing ginsenosides, especially for the rare types. Correspondingly, because of the unavailability of ginsenosides, the correlation of structures to anticancer activities is still not fully elucidated. In this study, three readily available ginsenosides (Rh1, Rg3 and Rh2) were used as the materials to produce three pairs of C-20(21)/C-20(22) double-bond isomers by chemical transformation. The effects of main factors on transformation of ginsenosides were investigated by ultra-performance liquid chromatography (UPLC) analysis. We explored the anticancer effects of 23 ginsenosides on six human cancer cell lines and uncovered the possible structural-activity relationships based on the number of sugar moieties, position of the sugar moieties, types of aglycone, position of double bond and stereoselectivity. An understanding of these relationships may provide insights into chemical and pharmacological approaches for developing novel cancer chemopreventive compounds.

## Results

### Transformation of ginsenosides

#### Effects of temperature on saponin transformation

The effects of temperature on ginsenosides transformation were investigated. Temperatures at 80°C, 100°C, 120°C, and 140°C were involved. The results showed that the ginsenoside products increased with increasing temperature, and achieved to the highest at 120°C. With a temperature of 140°C, concentrations of products were lower than those at 120°C (Fig. 2a).

#### Effects of time course on saponin transformation

The effects of heating time on the conversion of ginsenosides were also investigated (Fig. 2b). The contents of original ginsenosides declined gradually at 120°C from 1 to 6 h. Correspondingly, six newly occurring saponins increased gradually during the first 4 h and showed limited increase from 4–6 h. To improve the efficiency, 4 h was selected for ginsenosides transformation.

#### Effects of acid on saponin transformation

The pH environment is another determinant factor in the transformation process. To investigate the effects of acid concentration on saponin transformation, different concentrations of formic acid (0.01%, 0.1%, 0.5%, 2% and 5%) were employed (Fig. 2c). The results demonstrated that 0.01% of formic acid provided the highest yields.

#### Yields of rare ginsenosides

Preparation of ginsenoside Rk3/Rh4, Rk1/Rg5, and Rk2/Rh3 were achieved by dehydration at C-20 with 0.01% of formic acid at 120°C for 4 h. The reaction products were separated by UPLC (Fig. 3), and their structures were determined by ^13^C NMR spectroscopy (Supplementary Fig. S1–S6). The peaks with retention times of 4.7, 6.9, 7.0, 7.4, 8.3, 8.4, 8.7, 10.6, and 10.8 min corresponded to ginsenoside Rh1, Rk3, Rh4, Rg3, Rk1, Rg5, Rh2, Rk2, and Rh3, respectively. As shown in Table 1, from 100 mg of Rh1, 8.3 mg of Rk3 and 18.7 mg of Rh4 can be produced by acid transformation. Similarly, from 100 mg of Rg3, 7.4 mg of Rk1 and 15.1 mg of Rg5 can be produced. From 100 mg of Rh2, 8.3 mg of Rk2 and 12.7 mg of Rh3 can be generated. It seemed the yield of C-20(22) was higher than that of C-20(21) isomer.

### Structure-activity relationships of ginsenosides against cancer

A total of 23 ginsenosides were collected, from which the six rare ginsenosides produced by transformation were involved. Six human cancer cell lines were tested to uncover the structure-activity relationships of ginsenosides, including HCT-116, HepG2, MCF-7, Hela, PANC-1, and A549. The results from HepG2 cancer cells were used to elucidate structure-activity relationships of ginsenosides. Similar structure-activity relationships were obtained on other cancer cell lines shown in Supplementary Fig. S7.

#### Number of sugar moieties

The anticancer activities of ginsenosides with tetrasaccharide glycoside (ginsenosides Rb1, Rb2), trisaccharide glycoside (ginsenosides Re), disaccharide glycoside (ginsenosides Rk1, Rg1), monosaccharide glycoside (ginsenosides Rh2, Rh3, CK) and sapogenin (protopanaxadiol, protopanaxatriol) were compared. As shown in Fig. 4a, ginsenosides Rb1, Rb2, Re and Rd with three or four sugar moieties could almost not inhibit the growth of human cancer cells even at the concentration of 80 μM. In contrast, these ginsenosides promoted the proliferation at low concentrations. Rf and Rg1 having two sugar moieties showed weak cytotoxicity with IC_50_ values more than 80 μM. Ginsenosides with one sugar moiety could significantly inhibit the cell proliferation. Ginsenoside Rh2 and metabolite CK showed strongest cytotoxic effect among all the ginsenosides at a concentration at 10 μM. PPD and PPT, the aglycones of two dammarane types of ginsenoside, had medium cytotoxic effects with IC_50_ between 20–40 μM.

The results demonstrated that the activity of the ginsenosides ranked in a decreasing order as: monosaccharide glycoside > sapogenin > disaccharide glycoside > trisaccharide glycoside > tetrasaccharide glycoside. It implied that the sugar numbers is inversely related to the cytotoxic effects of ginsenosides.

#### Position of sugar

As shown in Fig. 4b, ginsenosides Rh1, Rh2 and F1 have one glucose but different linkage positions. Results showed that these ginsenosides exhibited differences in cytotoxic activities. For instance, ginsenoside Rh1, with sugar linkage at C-6, exerted stronger antiproliferative effect than ginsenoside F1 with sugar linkage at C-20. Ginsenoside Rh2, with a connection site at C-3, showed the strongest cytotoxic activities. Taken together, the cytotoxic potency of ginsenosides has been demonstrated to be in the order: C-3 > C-6 > C-20.

#### Different types of aglycone

PPD and PPT are the two major types of dammarane saponins. Compounds protopanaxadiol and protopanaxatriol, the aglycones of PPD-type and PPT-type ginsenosides, inhibited growth of cancer cells in a similar dose-dependent manner, but showed no significant difference. Rh2 and *S*-Rh1 along with CK and F1, possess the same sugar moiety but different types of aglycone. As shown in Fig. 4c, Rh1 (PPT-type) did not exert significant antiproliferative effects, while Rh2 (PPD-type) could inhibit the proliferation of 80% cells at the concentration of 10 μM. As an important ginsenoside of PPT-type, F1 could not inhibit the growth of cancer cells even at 80 μM, while CK (PPD-type) showed stronger anticancer activity than F1. Our observations suggest that different types of aglycone may make different contributions to cytotoxic activity, and it is possible that PPD-type ginsenosides have better anticancer activity.

#### Position of double bond

Ginsenosides Rk3/Rh4, Rk1/Rg5 and Rk2/Rh3 are the dehydroxylated products of Rh1, Rg3 and Rh2 at C-20, respectively. As shown in Fig. 4d, ginsenosides Rk1 and Rk3 with the double bond at C20-21 showed higher anticancer effects than Rg5 and Rh4 with double bond at C20-22. Ginsenoside Rh3 showed moderate cytotoxic effects from concentration range of 5 to 80 μM; while, Rk2 just showed some antiproliferative effect at a high concentration higher than 80 μM. In summary, ginsenosides with double bond at C20-21 exhibited more effective antitumor activities than those at C20-22.

#### Stereoselectivity of 20(R) and 20(S)

20(*R*) and 20(*S*) is a pair of stereoisomers with different position of C-20 hydroxyl in ginsenosides. 20(*S*)-OH is geometrically closer to the C-12 hydroxyl than 20(*R*)-OH. As shown in Fig. 4e, the stereostructure of the C-20 hydroxyl may influence the biological and pharmacological effects of ginsenosides. 20(*S*)-PPD showed stronger chemopreventive effect than its 20(*R*)-stereoisomer.

## Discussion

C-20 dehydroxylated dammarane-type ginsenosides, a group of rare ginsenosides with remarkable anticancer activities, mainly exist in steamed ginseng^21^. In this study, we aimed to prepare these rare ginsenosides by chemical transformation. Biotransformation methods were widely used to transfer original ginsenosides to deglycosylated products, such as Rd and CK^22^,^23^. Such biotransformation is not suitable to gain the dehydroxylated ones. Since C-20 rare dehydroxylated ginsenosides were present with high concentrations in ginseng after a steaming process, transformation by heating was considered in this study.

Ginsenosides could not be transformed under the neutral condition, and more by-products were produced when exposed to strong acids^24^. It was also known that ginsenosides tend to be transformed by deglycosylation in high acidic environment^12^. In this work, a low amount of acid was used to obtain dehydroxylated products, and also avoid deglycosylation reactions. This chemical transformation method was first applied to prepare rare dehydroxylated ginsenosides. After heating at 120°C for 4 h with 0.01% formic acid, ginsenoside Rh1, Rg3 and Rh2 were ultimately converted to three pairs of raw ginsenosides. The total transformation efficiency was more than 20%.

Due to the low specificity of chemical transformation, by-products produced during the conversion reduced the yield of the target products. To compare the chromatograms after transformation, two unknown by-products with larger polarity were observed. In addition, we found that acid treatments can induce epimerization reactions of ginsenosides, and their stereoisomers were further identified in reaction solution. Taken together, the existence of the by-products and the epimerization of the raw materials may result in the incompleteness of this conversion. Reduction of the side-reactions and ways to maximize the target-products, will be our emphases in further researches.

To the best of our knowledge, there is no systematic evaluation of the structure-activity relationship of the rare ginsenosides on the antiproliferative effect of cancer cells. Six human cancer cell lines were used to screen the activity of compounds and to uncover the structure-activity relationship of 23 ginsenosides (Supplementary Fig. S7). Sugar moiety is a key factor in moderating the activity of ginsenosides. The increasing number of sugar moieties reduces the cytotoxic effect of the compound. These observations imply that the anticancer effects of ginsenosides are inversely related to the number of sugar moieties, and get the strongest data when only one sugar is linked. As the hydrophobicity influences absorption of compounds, the presence of sugar moieties may reduce the hydrophobic character of the compounds and decrease their permeabilities to cell membranes^25^. This hydrophobic property is required to interact with specific membrane proteins or to pass into the nucleus, and may be the reason why their anticancer activities increase with the decrease of number of sugar moieties for ginsenosides. Besides, the anticancer activities of ginsenosides also depend on their hydrophilicity-lipophilicity balance.

Compared with C-3 linkage, sugar moieties at C-6 or C-20 increases steric hindrance for the entrance of ginsenosides and blocks compounds from extracellular binding to their targets, thus significantly reducing the anticancer activities of ginsenosides^26^. Previously, it was reported that 25-OH-PPD had stronger chemopreventive effect than 25-OH-PPT^27^, which is consistent with our observations that PPD-type ginsenosides have better anticancer activity.

Using the above optimal transformation conditions, rare ginsenosides with double bond at C-20 were obtained. We can speculate that the terminal double bond contribute more greatly to the antitumor activity in product ginsenosides in most cases. 20(*S*)-PPD and 20(*R*)-PPD, another pair of stereoisomers on the position of the C-20, have been compared for their anticancer activities. Wang et al. observed that 20(*S*)-PPD showed stronger chemopreventive effect than its 20(*R*)-stereoisomer^28^, which is in accordance with our speculation. The reason may be that C-20 hydroxyl group is spatially closer to the C-12 hydroxyl for 20(*S*) stereoisomer, and stereoselective interactions with lipid membranes may affect the antiproliferative activity of 20(*R*)/(*S*)-ginsenosides^29^. In summary, we determined that the ginsenosides (1) with one sugar or just aglycone; (2) with the sugar linkage at C-3; (3) PPD type of aglycone; (4) with double bond at C20-21; (5) stereoselectivity of 20(*S*) contribute most to their anticancer effects.

With respect to the potency of these ginsenosides, our results indicated that most of the rare ginsenosides have dominant chemotherapeutic activities, such as Rk1, Rk3, and Rh3. Further research in terms of pharmacological and mechanisms of activities are underway to bring clarity to these findings. Based on the uncovered structure-activity relationships, we will not only obtain useful information for modifying ginsenosides structure, but also select ginsenosides with superior anticancer activity for further testing and eventual drug development.

## Methods

### Materials

High performance liquid chromatography (HPLC) grade acetonitrile and methanol were obtained from Merck (Darmstadt, Germany). Formic acid with a purity of 99% was from ROE (Newark, New Castle, USA). Deionized water (18 MΩ cm^−1^) was prepared by distilled water through a Milli-Q system (Millipore, Bedford, MA, USA). Other reagents of analytical purity were utilized for sample preparation.

**Figure 1:**
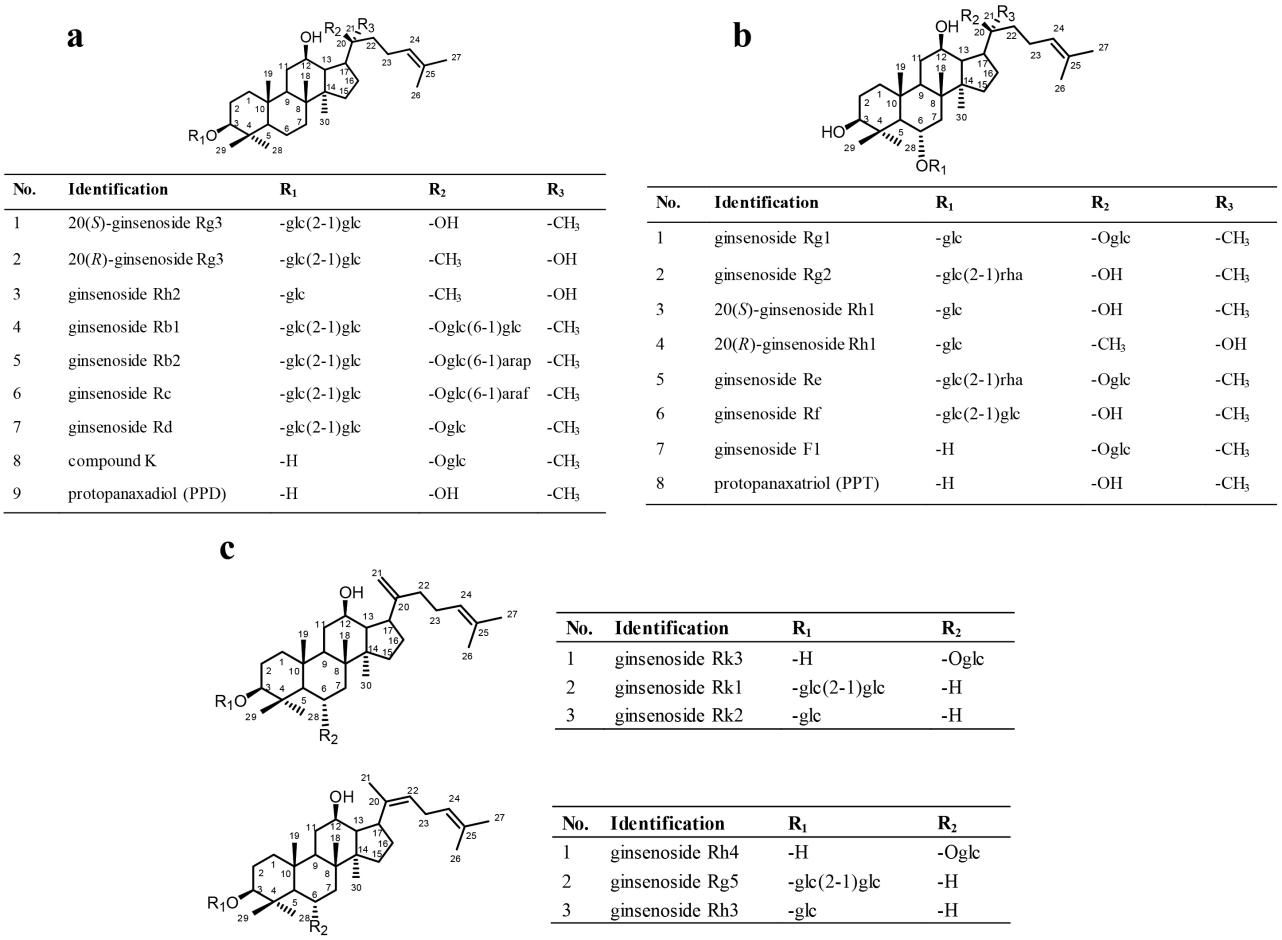
Chemical structures of the 23 studied ginsenosides. (a) protopanaxadiol (PPD)-type ginsneosides; (b) protopanaxatriol (PPT)-type ginsneosides; (c) rare ginsenosides. glc, *β*-D-glucose; rha, *α*-L-rhamnose; arap, *α*-L-arabinose (pyranose); araf, *α*-L-arabinose (furanose).

**Figure 2:**
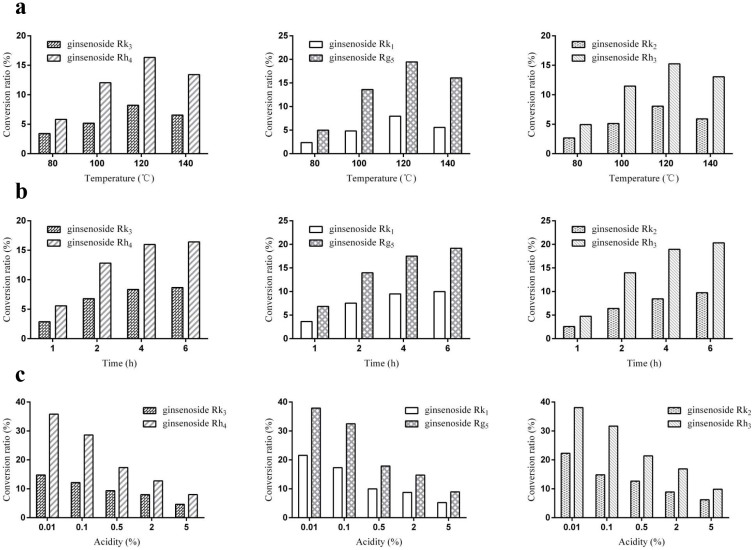
The effects of heating temperature, heating time and concentration of formic acid on transformation efficiencies. (a) Heating for 80°C, 100°C, 120°C, and 140°C; (b) heating for 1 h, 2 h, 4 h, and 6 h; (c) addition of 0.01%, 0.1%, 0.5%, 2%, and 5% of formic acid.

**Figure 3:**
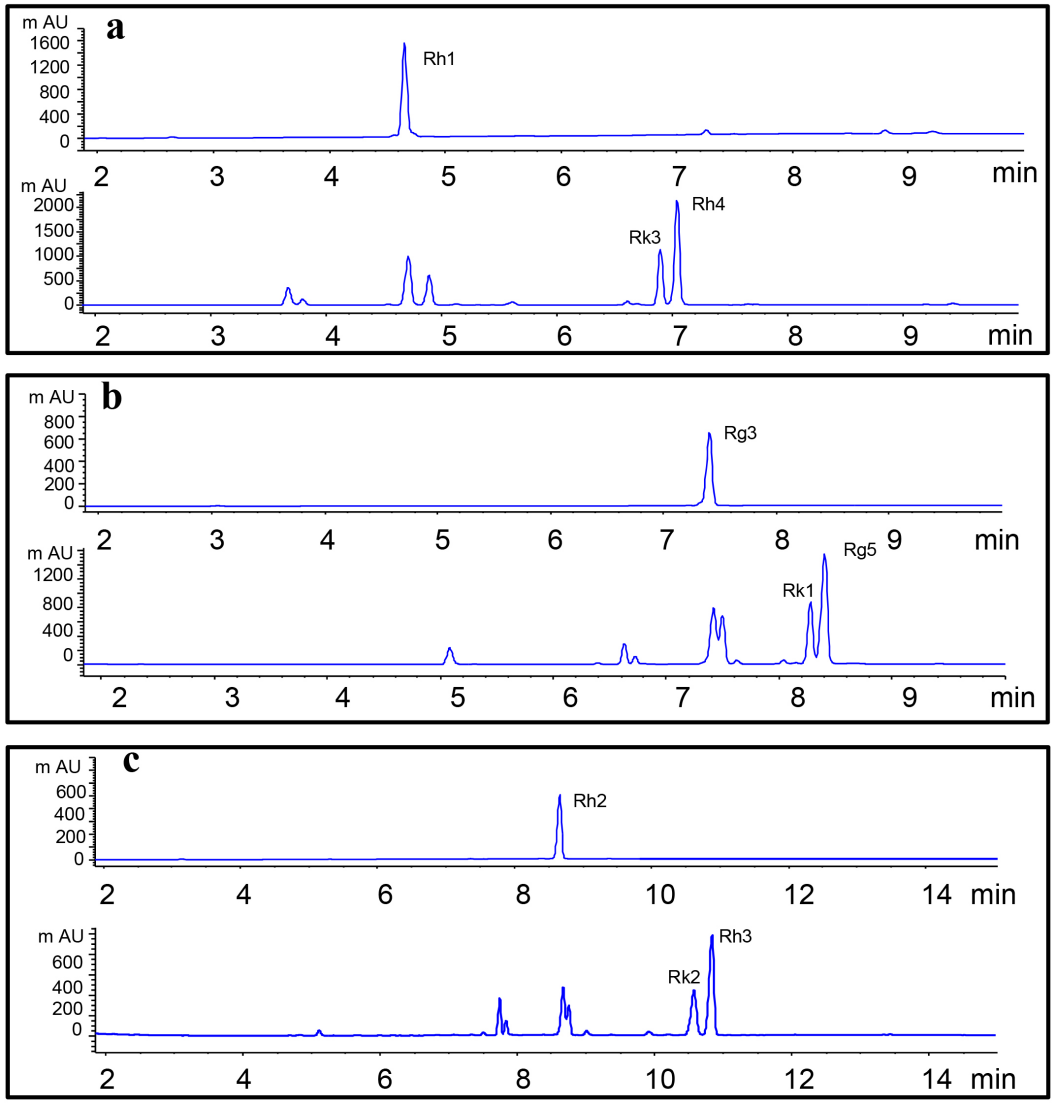
Monitoring of ginsenosides transformation by ultra-performance liquid chromatography with UV detection at 203 nm. (a) Transformation of ginsenoside Rh1 to Rk3 and Rh4; (b) ginsenoside Rg3 to Rk1 and Rg5; (c) ginsenoside Rh2 to Rk2 and Rh3. The chromatographic peaks were identified by comparing with the reference compounds.

**Figure 4:**
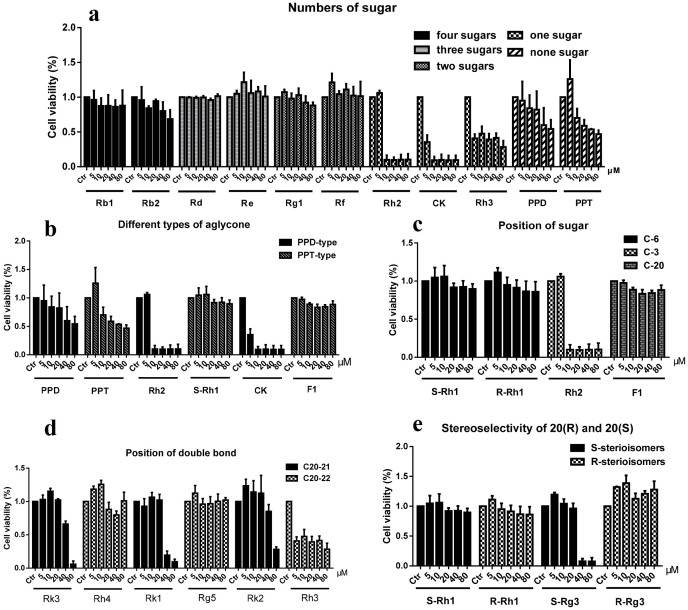
Antiproliferative effects of ginsenosides on human cancer HepG2 cells. (a) Comparison of ginsenosides with different sugar numbers; (b) ginsenosides with different linkage positions of sugars; (c) ginsenosides with different types of aglycones; (d) ginsenosides with different positions of double bonds; (e) ginsenosides with different stereoselectivities of 20(*R*) and 20(*S*). The anitproliferative effects were determined by the MTT assay and calculated by comparison with the control after exposure to 5, 10, 20, 40, and 80 μM of ginsenosides in each group for 24 h. The data were expressed as the mean ± SD.

Reference ginsenosides, Rb1, Rb2, Rc, Rd, Re, Rf, 20(*R*)/20(*S*)-Rh1, Rh2, Rg1, Rg2, 20(*R*)/20(*S*)-Rg3, F1, CK, protopanaxadiol and protopanaxatriol were purchased from Jilin University (Changchun, China). Their structures shown in Fig. 1 were further elucidated by spectroscopic methods (^1^H, ^13^C NMR and MS) with a purity of more than 95% for each compound in the authors' laboratory.

### Sample preparation

A sealed tube (Beijing Synthware Glass, China) was charged with reference ginsenosides (100 mg) and formic acid in 50 mL of 30% (*v/v*) methanol. The mixture was stirred at 80°C, 100°C, 120°C, and 140°C for 1, 2, 4, and 6 h, respectively. Then the reaction mixture was cooled at room temperature, evaporated to dryness, and redissolved with DMSO. The solution was centrifuged at 13,000 rpm for 10 min. Two microliters of the supernatants were subjected to UPLC for analysis.

### UPLC analysis

Analysis was performed on an Agilent 1290 Infinity UPLC system (Agilent Technology, Waldbronn, Germany) equipped with a binary pump, a diode-array detector, an auto sampler, and a thermostatically controlled column compartment. Samples were separated on an Agilent ZORBAX Extend-C18 column (2.1 mm × 50 mm, 1.8 μm) at 25°C using water (solvent A) and acetonitrile (solvent B). A gradient elution program was used according to the following profile: 20% B at 0–0.5 min, 20–26% B at 0.5–3 min, 26–38% B at 3–6 min, 38–55% B at 6–8 min, 55% B at 8–10 min, and 55–100% B at 10–15 min. The flow rate was kept at 0.8 mL/min and the optimum wavelength was set at 203 nm.

### HPLC preparation

An Agilent 1100 HPLC system (Agilent Technologies, Santa Clara, CA, USA) was utilized for ginsenosides preparation. The chromatographic separation was achieved on an Agilent ZORBAX SB-C18 semi-preparative column (9.4 mm × 250 mm, 5 μm) with a solvent flow rate of 1 mL/min at the temperature of 25°C. The mobile phase was composed of water (A) and acetonitrile (B). The products of ginsenoside Rh1, Rg3 and Rh2 were carried out with the constant elution of 50% B, 65% B and 85% B, respectively. The collected samples were evaporated under vacuum and lyophilized, then stored at 4°C.

### Cell culture

The cancer cell lines used in the study include human colon carcinoma (HCT-116), human liver carcinoma (HepG2), human cervical carcinoma (Hela), human breast adenocarcinoma (MCF-7), human pancreatic cancer (PANC-1), and human lung carcinoma (A549). All cells were purchased from American Type Culture Collection (Manassas, VA, USA) and grown in DMEM (Dulbecco's modified Eagles Medium). To ensure growth and viability of the cells, the medium was supplemented with 10% fetal bovine serum and 1% penicillin/streptomycin (Gibco, USA). The cells were cultured at 37°C in a humid incubator with 5% CO_2_.

### Cell proliferation assay

Assessment of cell proliferation was performed by MTT [3-(4,5-dimethylthiazol-2-yl)-2,5-diphenyl-2H-tetrazolium bromide] assay. Cells were seeded in 96-well plates at a density of 5 × 10^3^ per well, and allowed to attach for 24 h. Then they were treated with different concentrations of ginsenosides. Controls were exposed to culture medium with DMSO. After 24 h, 10 μL of MTT (5 mg/mL) was added to culture medium in each well and the plates were incubated for additional 4 h. Then the medium was removed and 200 μL DMSO was added to dissolve the blue formazan crystals converted from MTT. Finally, absorbance values were measured at 490 nm with Synergy 2 Multi-Mode Microplate Reader (BioTek Instruments Inc., USA). Results were expressed as a percentage versus control (vehicle set at 100%).

### Statistical analysis

Data were presented as means ± standard deviation (SD) from triplicate experiments performed in a parallel manner. Statistical analysis was conducted using Student's *t*-test.

## Author Contributions

L.W.Q. and P.L. designed the research; J.Y.W. and R.Z.G. conducted the chemical preparation; K.Q. and Y.J.Z. did the anticancer screening; K.Q. and Q.L. involved in data analysis, and co-wrote the manuscript; L.W.Q. and R.N.A. edited the manuscript. All authors approved the final version of the manuscript.

## Supplementary Material

Supplementary Information supplementary information

## Figures and Tables

**Table 1:** Transformation results of 100 mg ginsenosides with 0.01% formic acid at 120°C for 4 h

Material	Products	Isolated Yield	Purity
ginsenoside Rh_1_	ginsenoside Rk_3_	8.3%	99.0%
	ginsenoside Rh_4_	18.7%	90.5%
ginsenoside Rg_3_	ginsenoside Rk_1_	7.4%	93.5%
	ginsenoside Rg_5_	15.1%	97.2%
ginsenoside Rh_2_	ginsenoside Rk_2_	8.3%	98.3%
	ginsenoside Rh_3_	12.7%	99.7%

## References

[b1] Lee M. S., Yang E. J., Kim J. I. & Edzard E. Ginseng for cognitive function in Alzheimer's disease: a systematic review[J]. J Alzheimer's Dis, 18, 339–344 (2009). 19584437 10.3233/JAD-2009-1149

[b2] Lee H., Kim M., Shin S. S. & Yoon M. Ginseng treatment reverses obesity and related disorders by inhibiting angiogenesis in female db/db mice. J. Ethnopharmacol. 155, 1342–1352 (2014). 25072361 10.1016/j.jep.2014.07.034

[b3] Shishtar E., Jovanovski E., Jenkins A. & Vuksan V. Effects of Korean White Ginseng (*Panax Ginseng C. A. Meyer*) on Vascular and Glycemic Health in Type 2 Diabetes: Results of a Randomized, Double Blind, Placebo-controlled, Multiple-crossover, Acute Dose Escalation Trial. Clin. Nutr. Res. 3, 89–97 (2014). 25136536 10.7762/cnr.2014.3.2.89 PMC4135246

[b4] Jia L., Zhao Y. & Liang X. J. Current evaluation of the millennium phytomedicine- ginseng (II): Collected chemical entities, modern pharmacology, and clinical applications emanated from traditional Chinese medicine. Curr. Med. Chem. 16, 2924–2942 (2009). 19689273 10.2174/092986709788803204 PMC2754208

[b5] Qi L. W., Wang C. Z. & Yuan C. S. Isolation and analysis of ginseng: advances and challenges. Nat. Prod. Rep. 28, 467–495 (2011). 21258738 10.1039/c0np00057d PMC3056508

[b6] Chung K. S. *et al.* Ginsenoside Rh2 induces cell cycle arrest and differentiation in human leukemia cells by upregulating TGF-beta expression. Carcinogenesis. 34, 331–340 (2013). 23125221 10.1093/carcin/bgs341

[b7] Li J. *et al.* Ginsenoside Rg1 induces apoptosis through inhibition of the EpoR-mediated JAK2/STAT5 signalling pathway in the TF-1/Epo human leukemia cell line. Asian. Pac. J. Cancer. Prev. 15, 2453–2459 (2014). 24761846 10.7314/apjcp.2014.15.6.2453

[b8] Zeng D., Wang J., Kong P., Chang C. & Li J. Ginsenoside Rg3 inhibits HIF-1alpha and VEGF expression in patient with acute leukemia via inhibiting the activation of PI3K/Akt and ERK1/2 pathways. Int. J. Clin. Exp. Pathol. 7, 2172–2178 (2014). 24966925 PMC4069960

[b9] Mao Q., Zhang P. H., Wang Q. & Li S. L. Ginsenoside F(2) induces apoptosis in humor gastric carcinoma cells through reactive oxygen species-mitochondria pathway and modulation of ASK-1/JNK signaling cascade in vitro and in vivo. Phytomedicine. 21, 515–522 (2014). 24252332 10.1016/j.phymed.2013.10.013

[b10] Shin Y. M., Jung H. J., Choi W. Y. & Lim C. J. Antioxidative, anti-inflammatory, and matrix metalloproteinase inhibitory activities of 20(S)-ginsenoside Rg3 in cultured mammalian cell lines. Mol. Biol. Rep. 40, 269–279 (2013). 23054007 10.1007/s11033-012-2058-1

[b11] Yu H. *et al.* Development of liposomal Ginsenoside Rg3: formulation optimization and evaluation of its anticancer effects. Int. J. Pharm. 450, 250–258 (2013). 23628402 10.1016/j.ijpharm.2013.04.065

[b12] Kim M. H. *et al.* The changes of ginsenoside patterns in red ginseng processed by organic acid impregnation pretreatment. J. Gins. Res. 35, 497–503 (2011). 10.5142/jgr.2011.35.4.497 PMC3659558 23717097

[b13] Qi L. W. *et al.* Diagnostic ion filtering to characterize ginseng saponins by rapid liquid chromatography with time-of-flight mass spectrometry. J. Chromatogr. A. 1230, 93–99 (2012). 22349142 10.1016/j.chroma.2012.01.079

[b14] Kim B. H. *et al.* Biotransformation of Korean *Panax ginseng* by Pectinex. Biol. Pharm. Bull. 29, 2472–2478 (2006). 17142984 10.1248/bpb.29.2472

[b15] Choi J. R. *et al.* Metabolic activities of ginseng and its constituents, ginsenoside Rb1 and Rg1, by human intestinal microflora. J. Gins. Res. 35, 301–307 (2011). 10.5142/jgr.2011.35.3.301 PMC3659535 23717073

[b16] Chang K. H., Jo M. N., Kim K. T. & Paik H. D. Evaluation of glucosidases of *Aspergillus niger* strain comparing with other glucosidases in transformation of ginsenoside Rb1 to ginsenosides Rg3. J. Gins. Res. 38, 47–51 (2014). 10.1016/j.jgr.2013.11.008 PMC3915331 24558310

[b17] Wang J. R. *et al.* Transformation of ginsenosides from notoginseng by artificial gastric juice can increase cytotoxicity toward cancer cells. J. Agric. Food Chem. 62, 2558–2573 (2014). 24555416 10.1021/jf405482s

[b18] Yan X. *et al.* Production of bioactive ginsenoside compound K in metabolically engineered yeast. Cell Res. 24, 770–773 (2014). 24603359 10.1038/cr.2014.28 PMC4042165

[b19] Kim Y. J. *et al.* Anti-tumor activity of the ginsenoside Rk1 in human hepatocellular carcinoma cells through inhibition of telomerase activity and induction of apoptosis. Biol. Pharm. Bull. 31, 826–830 (2008). 18451501 10.1248/bpb.31.826

[b20] Ko H., Kim Y. J., Park J. S., Park J. H. & Yang H. O. Autophagy inhibition enhances apoptosis induced by ginsenoside Rk1 in hepatocellular carcinoma cells. Biosci. Biotechnol. Biochem. 73, 2183–2189 (2009). 19809182 10.1271/bbb.90250

[b21] Sun S. *et al.* Red notoginseng: higher ginsenoside content and stronger anticancer potential than Asian and American ginseng. Food Chem. 125, 1299–1305 (2011). 21344064 10.1016/j.foodchem.2010.10.049 PMC3041968

[b22] Zhao X. *et al.* Highly selective biotransformation of ginsenoside Rb1 to Rd by the phytopathogenic fungus *Cladosporium fulvum* (*syn. Fulvia fulva*). J. Ind. Microbiol. Biot. 36, 721–726 (2009). 10.1007/s10295-009-0542-y 19229572

[b23] Kim S. H. *et al.* Enzymatic Transformation of Ginsenoside Rb1 by *Lactobacillus pentosus* Strain 6105 from Kimchi. J. Gins. Res. 36, 291–297 (2012). 10.5142/jgr.2012.36.3.291 PMC3659591 23717130

[b24] Bae E. A., Han M. J., Kim E. J. & Kim D. H. Transformation of ginseng saponins to ginsenoside Rh2 by acids and human intestinal bacteria and biological activities of their transformants. Arch. Pharm. Res. 27, 61–67 (2004). 14969341 10.1007/BF02980048

[b25] Jia L. & Zhao Y. Current evaluation of the millennium phytomedicine--ginseng (I): etymology, pharmacognosy, phytochemistry, market and regulations. Curr. Med. Chem. 16, 2475–2484 (2009). 19601793 10.2174/092986709788682146 PMC2752963

[b26] Li W. *et al.* Anti-androgen-independent prostate cancer effects of ginsenoside metabolites in vitro: mechanism and possible structure-activity relationship investigation. Arch. Pharm. Res. 32, 49–57 (2009). 19183876 10.1007/s12272-009-1117-1

[b27] Chen R. J., Chung T. Y., Li F. Y., Lin N. H. & Tzen J. T. Effect of sugar positions in ginsenosides and their inhibitory potency on Na+/K+-ATPase activity. Acta. Pharmacol. Sin. 30, 61–69 (2009). 19060914 10.1038/aps.2008.6 PMC4006530

[b28] Wang W. *et al.* In vitro anti-cancer activity and structure-activity relationships of natural products isolated from fruits of *Panax ginseng*. Cancer Chemoth. Pharm. 59, 589–601 (2007). 10.1007/s00280-006-0300-z 16924497

[b29] Qi L. W., Wang C. Z. & Yuan C. S. American ginseng: potential structure-function relationship in cancer chemoprevention. Biochem. Pharmacol. 80, 947–954 (2010). 20599804 10.1016/j.bcp.2010.06.023

