# Supplementary material for: Rapid preparation of rare ginsenosides by acid transformation and their structure-activity relationships against cancer cells
Source: Sci Rep. 2015 Feb 26;5:8598. doi: 10.1038/srep08598 (PMC4341195; doi:10.1038/srep08598)
Supplement: Supplementary Information [file srep08598-s1.pdf]

# Supplementary Information

## Rapid preparation of rare ginsenosides by acid transformation and their structure-activity relationships against cancer cells

Kai Quan[1], Qun Liu[1], Jin-Yi Wan[1], Yi-Jing Zhao, Ru-Zhou Guo, Raphael N Alolga, Ping Li [*], Lian-Wen Qi [*]

*State Key Laboratory of Natural Medicines, China Pharmaceutical University, No. 24 Tongjia Lane, Nanjing 210009, China*

* Corresponding authors. Tel./fax: +86 25 83271379.

E-mail addresses: Qilw@cpu.edu.cn (L.-W. Qi) or Liping2004@126.com (P. Li)

[1] These authors contributed equally to this work.

**Structure identification of dehydroxylated products**

**1.1 Structure identification of compound 1.**

Compound 1 was obtained as a white powder and can be soluble in methanol. The TOF-MS spectrum showed an $[M-H]^-$ ion at *m/z* 619.4227, and together with an $[M+HCOO]^-$ ion at *m/z* 665.4283, this indicated a molecular formula of $C_{36}H_{60}O_8$.

$^{13}$C-NMR (500 MHz, $C_5D_5N$) $\delta$: 40.040 (C-1), 28.430 (C-2), 79.097 (C-3), 41.810 (C-4), 61.965 (C-5), 80.519 (C-6), 45.866 (C-7), 40.860 (C-8), 51.160 (C-9), 40.249 (C-10), 33.227 (C-11), 72.973 (C-12), 52.630 (C-13),51.652 (C-14), 33.011 (C-15), 31.202 (C-16), 48.754 (C-17), 17.863 (C-18), 18.227 (C-19), 155.981 (C-20), 108.655 (C-21), 34.273 (C-22), 27.574 (C-23), 125.837 (C-24), 131.691 (C-25), 26.214 (C-26), 17.863 (C-27), 32.200 (C-28), 16.828 (C-29), 17.274 (C-30), 106.500 (C-1'), 75.963 (C-2'), 80.119 (C-3'), 72.411 (C-4'), 78.582 (C-5'), 63.635 (C-6'). These results are in agreement with data of Rk3 from literature[1]. Compound 1 was identified as Rk3.

**1.2 Structure identification of compound 2.**

Compound 2 was a white powder and can be soluble in methanol. It had the molecular formula $C_{36}H_{60}O_8$ determined from the quasi-molecular peaks at *m/z* 619.4219 $[M-H]^-$ and *m/z* 665.4274 $[M+HCOO]^-$ in its TOF-MS spectrum.

$^{13}$C-NMR (500 MHz, $C_5D_5N$) $\delta$: 39.320 (C-1), 27.717 (C-2), 78.378 (C-3), 40.146 (C-4), 61.246 (C-5), 79.812 (C-6), 45.154 (C-7), 41.162 (C-8), 50.371 (C-9), 39.559 (C-10), 32.054 (C-11), 72.373 (C-12), 50.512 (C-13),50.627 (C-14), 32.335

(C-15), 28.573 (C-16), 50.193 (C-17), 17.182 (C-18), 17.502 (C-19), 139.926 (C-20), 12.892 (C-21), 123.052 (C-22), 27.217 (C-23), 123.448 (C-24), 131.032 (C-25), 25.428 (C-26), 17.502 (C-27), 31.497 (C-28), 16.124 (C-29), 16.609 (C-30), 105.792 (C-1’), 75.250 (C-2’), 79.418 (C-3’), 71.698 (C-4’), 77.878 (C-5’), 62.934 (C-6’). These results are in agreement with data of Rh4 from literatures[1,2]. Compound 2 was identified as Rh4.

**1.3 Structure identification of compound 3**

Compound 3 was a white powder and can be soluble in methanol. It had the molecular formula $C_{42}H_{70}O_{12}$ deduced from an $[M\text{-}H]^-$ molecular peak at *m/z* 765.4782 and an $[M\text{+}HCOO]^-$ molecular peak at *m/z* 811.4829 in its negative TOF-MS.

$^{13}C$-NMR (500MHz, $C_5D_5N$) $\delta$: 39.801 (C-1), 27.260 (C-2), 89.430 (C-3), 40.215 (C-4), 56.937 (C-5), 18.949 (C-6), 35.860 (C-7), 40.717 (C-8), 48.728 (C-9), 37.539 (C-10), 33.092 (C-11), 72.971 (C-12), 53.005 (C-13),51.707 (C-14), 33.174 (C-15), 31.254 (C-16), 51.359 (C-17), 17.078 (C-18), 17.078 (C-19), 156.074 (C-20), 108.638 (C-21), 34.395 (C-22), 27.589 (C-23), 125.823 (C-24), 131.693 (C-25), 26.209 (C-26), 18.216 (C-27), 28.614 (C-28), 17.477 (C-29), 17.477 (C-30), 105.584 (C-1’), 84.028 (C-2’), 78.670 (C-3’), 72.172 (C-4’), 78.471 (C-5’), 63.280 (C-6’), 106.560 (C-1”), 77.597 (C-2”), 78.854 (C-3”), 72.249 (C-4”), 78.555 (C-5”), 63.392 (C-6”). These results are in agreement with data of Rk1 from literature[1]. Compound 3 was identified as Rk1.

**1.4 Structure identification of compound 4**

Compound 4 was obtained as a white powder and can be soluble in methanol. The TOF-MS spectrum showed an $[M\text{-}H]^-$ ion at *m/z* 765.4806, and together with an $[M+HCOO]^-$ ion at *m/z* 811.4861, this indicated a molecular formula of $C_{42}H_{70}O_{12}$.

$^{13}$C-NMR (500MHz, $C_5D_5N$) $\delta$: 39.319 (C-1), 26.778 (C-2), 88.952 (C-3), 39.735 (C-4), 56.450 (C-5), 18.465 (C-6), 35.381 (C-7), 40.294 (C-8), 50.798 (C-9), 37.064 (C-10), 32.225 (C-11), 72.627 (C-12), 50.459 (C-13), 51.070 (C-14), 32.225 (C-15), 28.839 (C-16), 50.922 (C-17), 16.609 (C-18), 16.609 (C-19), 140.213 (C-20), 13 (C-21), 123.305 (C-22), 27.461 (C-23), 123.701 (C-24), 131.280 (C-25), 25.673 (C-26), 17.717 (C-27), 28.137 (C-28), 17.046 (C-29), 17.046 (C-30), 105.114 (C-1'), 83.524 (C-2'), 78.208 (C-3'), 71.682 (C-4'), 77.981 (C-5'), 62.777 (C-6'), 105.114 (C-1''), 77.124 (C-2''), 78.366 (C-3''), 71.744 (C-4''), 78.088 (C-5''), 62.899 (C-6''). These results are in agreement with data of Rg5 from literature[1]. Compound 4 was identified as Rg5.

**1.5 Structure identification of compound 5**

Compound 5 was a white powder and can be soluble in methanol. It had the molecular formula $C_{36}H_{60}O_7$ determined from the quasi-molecular peaks at *m/z* 603.4283 $[M\text{-}H]^-$ and *m/z* 645.4348 $[M+HCOO]^-$ in its TOF-MS spectrum.

$^{13}$C-NMR (500MHz, $C_5D_5N$) $\delta$: 39.812 (C-1), 27.254 (C-2), 89.295 (C-3), 40.206 (C-4), 56.949 (C-5), 18.988 (C-6), 35.897 (C-7), 40.829 (C-8), 51.378 (C-9),

37.627 (C-10), 33.095 (C-11), 72.965 (C-12), 53.017 (C-13),51.728 (C-14), 34.207 (C-15), 28.896 (C-16), 48.752 (C-17), 16.364 (C-18), 16.948 (C-19), 140.313 (C-20), 125.049 (C-21), 33.207 (C-22), 27.605 (C-23), 125.845 (C-24), 140.313 (C-25), 26.229 (C-26), 18.253 (C-27), 28.673 (C-28), 17.290 (C-29), 17.509 (C-30), 107.434 (C-1'), 76.313 (C-2'), 79.279 (C-3'), 72.453 (C-4'), 78.833 (C-5'), 63.637 (C-6'). These results are in agreement with data of Rk2 from literature[1]. Compound 5 was identified as Rk2.

**1.6 Structure identification of compound 6**

Compound 6 was a white powder and can be soluble in methanol. It had the molecular formula $C_{36}H_{60}O_7$ deduced from an $[M-H]^-$ molecular peak at *m/z* 603.4254 and an $[M+HCOO]^-$ molecular peak at *m/z* 645.4307 in its negative TOF-MS.

$^{13}$C-NMR (500MHz, $C_5D_5N$) $\delta$: 39.802 (C-1), 28.657 (C-2), 89.282 (C-3), 40.796 (C-4), 56.937 (C-5), 16.936 (C-6), 35.859 (C-7), 40.190 (C-8), 51.288 (C-9), 37.599 (C-10), 32.712 (C-11), 73.087 (C-12), 50.945 (C-13), 51.564 (C-14), 33.135 (C-15), 27.244 (C-16), 51.400 (C-17), 18.694 (C-18), 17.271 (C-19), 140.693 (C-20), 13.654 (C-21), 124.299 (C-22), 27.947 (C-23), 123.534 (C-24), 131.767 (C-25), 26.149 (C-26), 18.196 (C-27), 29.323 (C-28), 16.340 (C-29), 17.547 (C-30), 107.435 (C-1'), 76.302 (C-2'), 79.266 (C-3'), 72.450 (C-4'), 78.832 (C-5'), 63.634 (C-6'). These results are in agreement with data of Rh3 from literature[1]. Compound 6 was identified as Rh3.

## Reference


1 Park, I. H. *et al.* Three new dammarane glycosides from heat processed ginseng. *Arch. Pharm. Res.* **25**, 428-432 (2002).

2 Baek, N. I. *et al.* Ginsenoside Rh4, a genuine dammarane glycoside from Korean red ginseng. *Planta Med.* **62**, 86-87 (1996).

**Figure Legends**

**Figure S1 $^{13}$C-NMR spectra of compound 1.**

**Figure S2 $^{13}$C-NMR spectra of compound 2.**

**Figure S3 $^{13}$C-NMR spectra of compound 3.**

**Figure S4 $^{13}$C-NMR spectra of compound 4.**

**Figure S5 $^{13}$C-NMR spectra of compound 5.**

**Figure S6 $^{13}$C-NMR spectra of compound 6.**

**Figure S7 Antiproliferation effect of 23 ginsenosides on six human cancer cells (HCT-116, HepG2, MCF-7, Hela, PANC-1, and A549).** The antiproliferative effect was determined by the MTT assay and calculated by comparison with the control after exposure to 5, 10, 20, 40, and 80 μM concentration of ginsenosides for 24 h. The data are expressed as the mean ± SD.

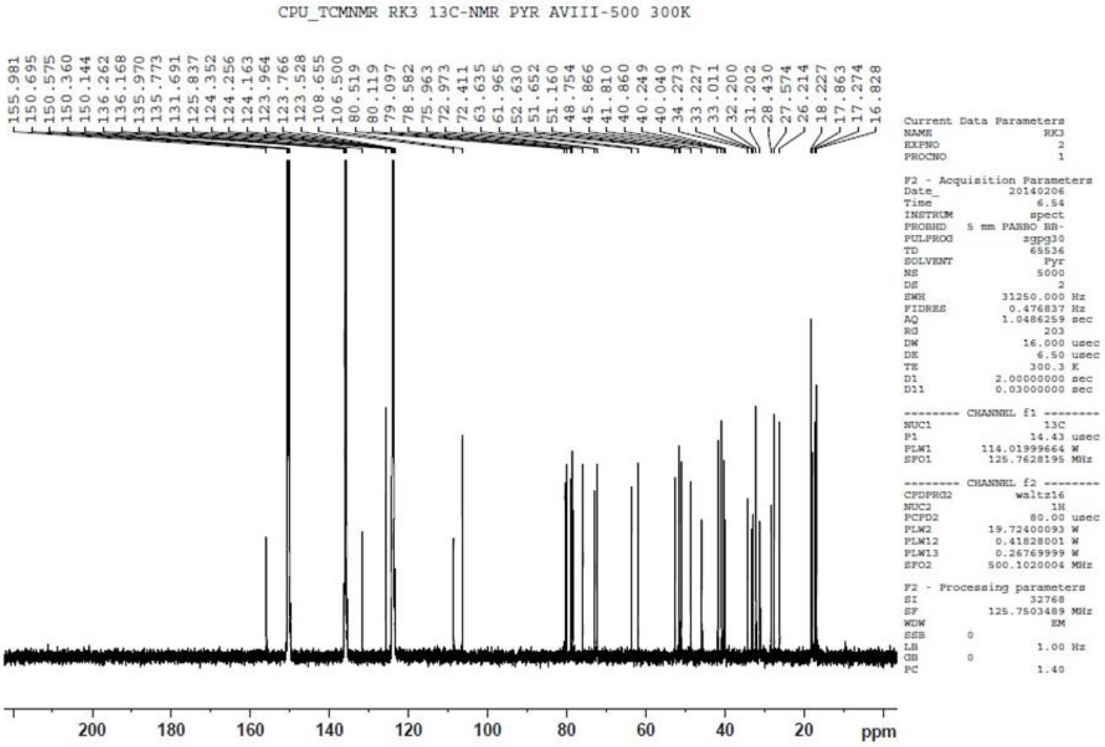
CPU_TCMNMR RK3 13C-NMR PYR AVIII-500 300K
155.981
150.695
150.575
150.360
150.144
136.262
136.168
135.970
135.773
131.691
125.837
124.352
124.256
124.163
123.964
123.766
123.528
108.655
106.500
80.519
80.119
79.097
78.582
75.963
72.973
72.411
63.635
61.965
52.630
51.652
51.160
48.754
45.866
41.810
40.860
40.249
40.040
34.273
33.227
33.011
32.200
31.202
28.430
27.574
26.214
18.227
17.863
17.274
16.828
200
180
160
140
120
100
80
60
40
20
ppm
Current Data Parameters
NAME RK3
EXPNO 2
PROCNO 1
F2 - Acquisition Parameters
Date_ 20140206
Time 6.54
INSTRUM spect
PROBHD 5 mm PABBO BB-
PULPROG zgpg30
TD 65536
SOLVENT Pyr
NS 5000
DS 2
SWH 31250.000 Hz
FIDRES 0.476837 Hz
AQ 1.0486259 sec
RG 203
DW 16.000 usec
DE 6.50 usec
TE 300.3 K
D1 2.00000000 sec
D11 0.03000000 sec
======== CHANNEL f1 ========
NUC1 13C
P1 14.43 usec
PLW1 114.01999664 W
SFO1 125.7628195 MHz
======== CHANNEL f2 ========
CPDPRG2 waltz16
NUC2 1H
PCPD2 80.00 usec
PLW2 19.72400093 W
PLW12 0.41828001 W
PLW13 0.26769999 W
SFO2 500.1020004 MHz
F2 - Processing parameters
SI 32768
SF 125.7503489 MHz
WDW EM
SSB 0
LB 1.00 Hz
GB 0
PC 1.40

**Figure S1**

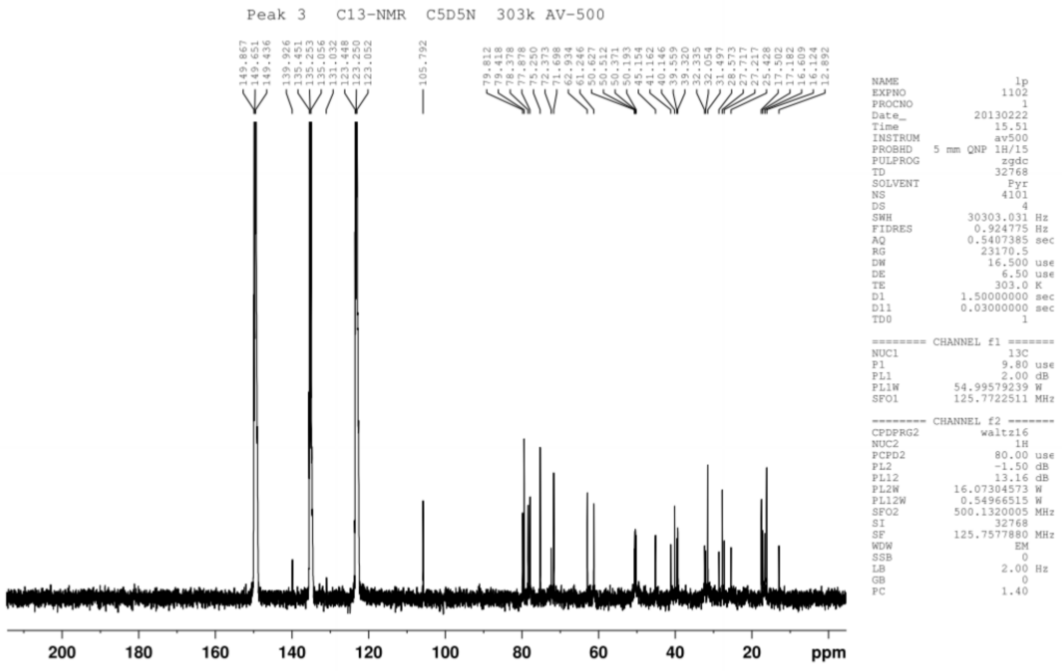
Peak 3 C13-NMR C5D5N 303k AV-500
149.867
149.651
149.436
139.926
135.451
135.253
135.056
131.032
123.448
123.250
123.052
105.792
79.812
79.418
78.378
77.878
75.250
72.373
71.698
62.934
61.246
50.627
50.512
50.371
50.193
45.154
41.162
40.146
39.559
39.320
32.335
32.054
31.497
28.573
27.717
27.217
25.428
17.502
17.182
16.609
16.124
12.892
NAME 1p
EXPNO 1102
PROCNO 1
Date_ 20130222
Time 15.51
INSTRUM av500
PROBHD 5 mm QNP 1H/15
PULPROG zgdc
TD 32768
SOLVENT Pyr
NS 4101
DS 4
SWH 30303.031 Hz
FIDRES 0.924775 Hz
AQ 0.5407385 sec
RG 23170.5
DW 16.500 use
DE 6.50 use
TE 303.0 K
D1 1.50000000 sec
D11 0.03000000 sec
TD0 1
======== CHANNEL f1 =======
NUC1 13C
P1 9.80 use
PL1 2.00 dB
PL1W 54.99579239 W
SFO1 125.7722511 MHz
======== CHANNEL f2 =======
CPDPRG2 waltz16
NUC2 1H
PCPD2 80.00 use
PL2 -1.50 dB
PL12 13.16 dB
PL2W 16.07304573 W
PL12W 0.54966515 W
SFO2 500.1320005 MHz
SI 32768
SF 125.7577880 MHz
WDW EM
SSB 0
LB 2.00 Hz
GB 0
PC 1.40
200
180
160
140
120
100
80
60
40
20
ppm

**Figure S2**

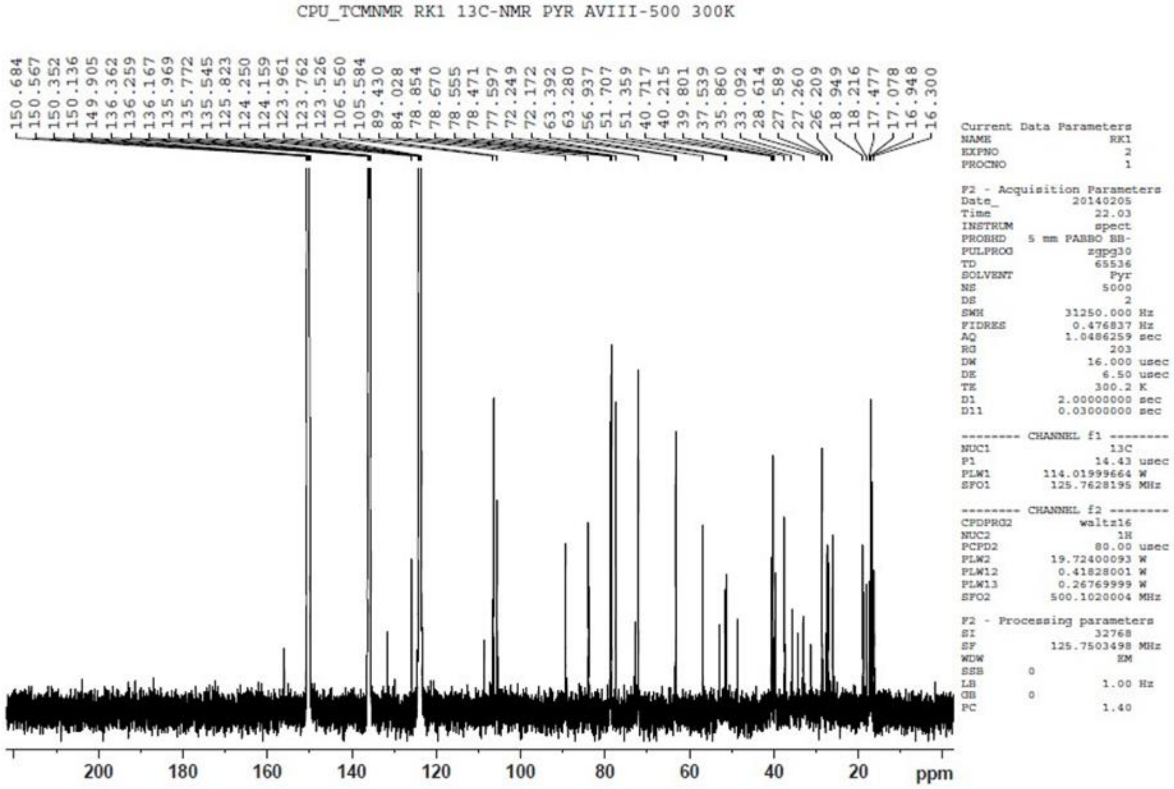

CPU_TCMNMR RK1 13C-NMR PYR AVIII-500 300K
150.684
150.567
150.352
150.136
149.905
136.362
136.259
136.167
135.969
135.772
135.545
125.823
124.250
124.159
123.961
123.762
123.526
106.560
105.584
89.430
84.028
78.854
78.670
78.555
78.471
77.597
72.249
72.172
63.392
63.280
56.937
51.707
51.359
40.717
40.215
39.801
37.539
35.860
33.092
28.614
27.589
27.260
26.209
18.949
18.216
17.477
17.078
16.948
16.300
Current Data Parameters
NAME RK1
EXPNO 2
PROCNO 1
F2 - Acquisition Parameters
Date_ 20140205
Time 22.03
INSTRUM spect
PROBHD 5 mm PABBO BB-
PULPROG zgpg30
TD 65536
SOLVENT Pyr
NS 5000
DS 2
SWH 31250.000 Hz
FIDRES 0.476837 Hz
AQ 1.0486259 sec
RG 203
DW 16.000 usec
DE 6.50 usec
TE 300.2 K
D1 2.00000000 sec
D11 0.03000000 sec
======== CHANNEL f1 ========
NUC1 13C
P1 14.43 usec
PLW1 114.01999664 W
SFO1 125.7628195 MHz
======== CHANNEL f2 ========
CPDPRG2 waltz16
NUC2 1H
PCPD2 80.00 usec
PLW2 19.72400093 W
PLW12 0.41828001 W
PLW13 0.26769999 W
SFO2 500.1020004 MHz
F2 - Processing parameters
SI 32768
SF 125.7503498 MHz
WDW EM
SSB 0
LB 1.00 Hz
GB 0
PC 1.40
200
180
160
140
120
100
80
60
40
20
ppm


**Figure S3**

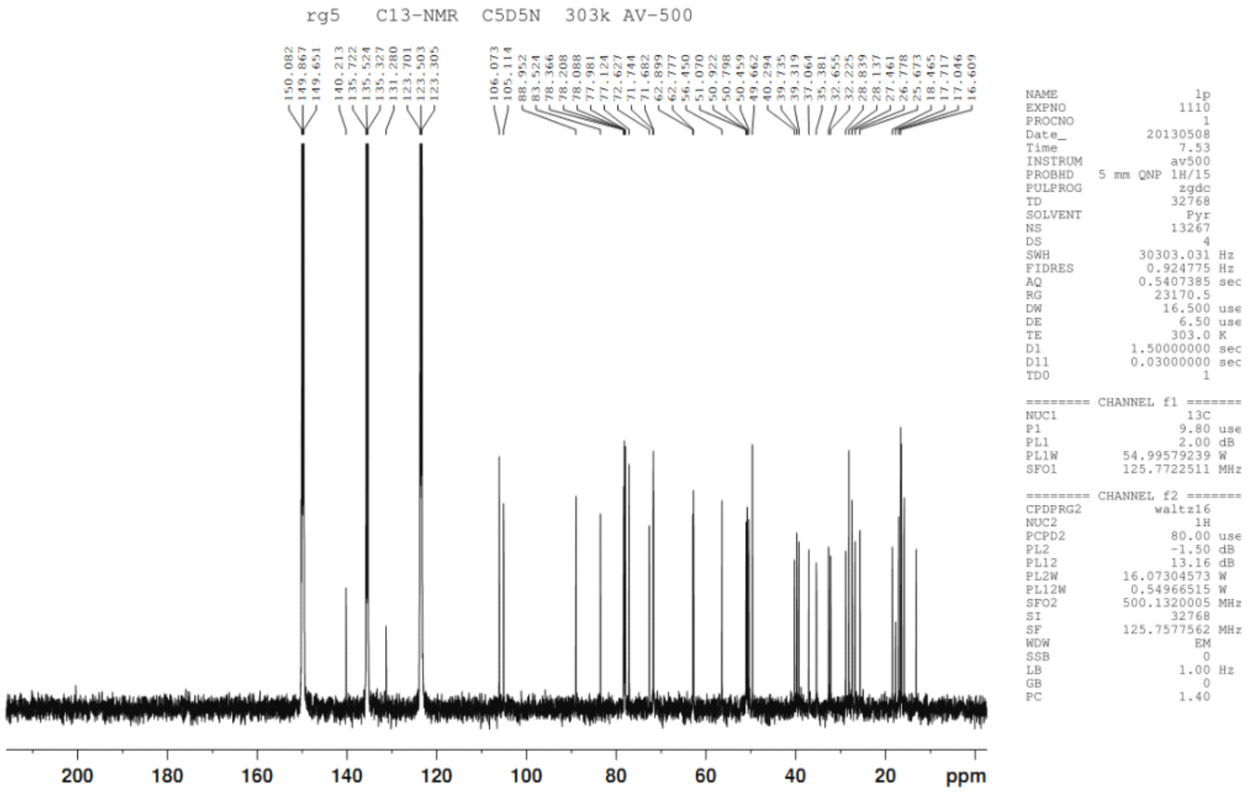
rg5 C13-NMR C5D5N 303k AV-500
NAME 1p
EXPNO 1110
PROCNO 1
Date_ 20130508
Time 7.53
INSTRUM av500
PROBHD 5 mm QNP 1H/15
PULPROG zgdc
TD 32768
SOLVENT Pyr
NS 13267
DS 4
SWH 30303.031 Hz
FIDRES 0.924775 Hz
AQ 0.5407385 sec
RG 23170.5
DW 16.500 use
DE 6.50 use
TE 303.0 K
D1 1.50000000 sec
D11 0.03000000 sec
TD0 1
======== CHANNEL f1 =======
NUC1 13C
P1 9.80 use
PL1 2.00 dB
PL1W 54.99579239 W
SFO1 125.7722511 MHz
======== CHANNEL f2 =======
CPDPRG2 waltz16
NUC2 1H
PCPD2 80.00 use
PL2 -1.50 dB
PL12 13.16 dB
PL2W 16.07304573 W
PL12W 0.54966515 W
SFO2 500.1320005 MHz
SI 32768
SF 125.7577562 MHz
WDW EM
SSB 0
LB 1.00 Hz
GB 0
PC 1.40
200 180 160 140 120 100 80 60 40 20 ppm

**Figure S4**

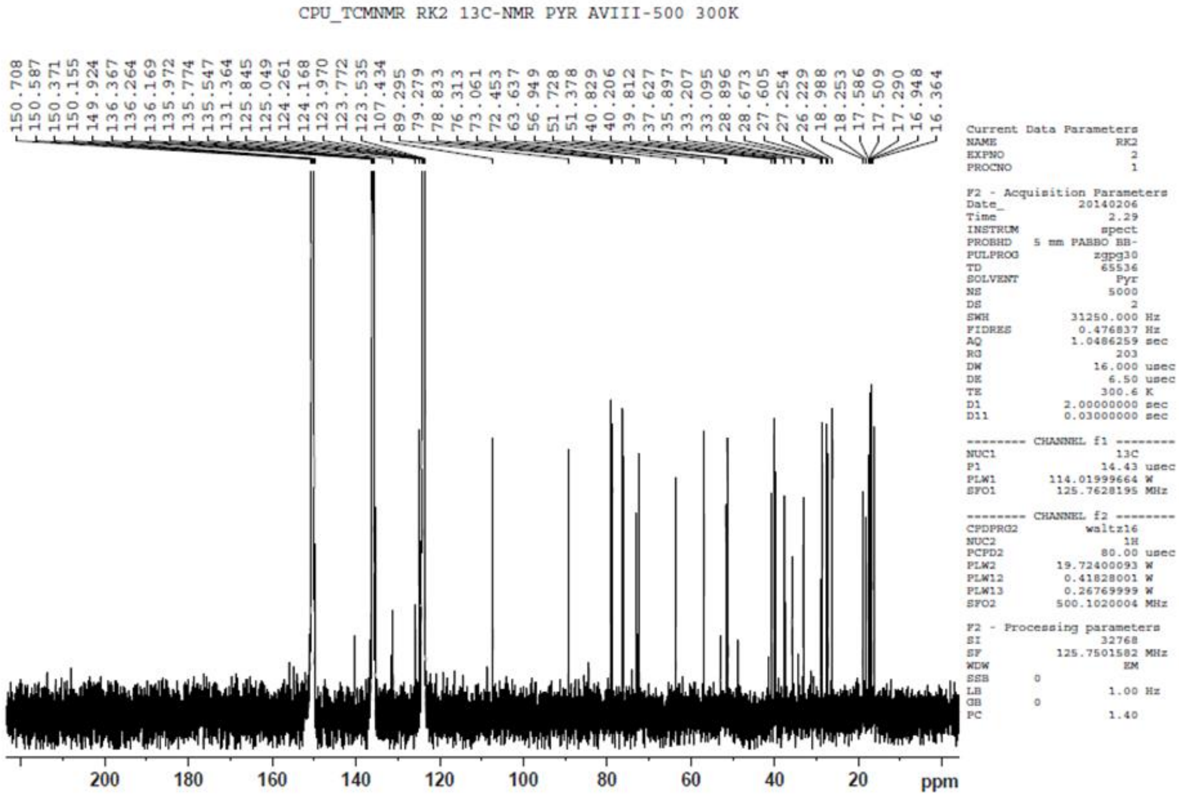
CPU_TCMNMR RK2 13C-NMR PYR AVIII-500 300K
150.708
150.587
150.371
150.155
149.924
136.367
136.264
136.169
135.972
135.774
135.547
131.364
125.845
125.049
124.261
124.168
123.970
123.772
123.535
107.434
89.295
79.279
78.833
76.313
73.061
72.453
63.637
56.949
51.728
51.378
40.829
40.206
39.812
37.627
35.897
33.207
33.095
28.896
28.673
27.254
27.605
26.229
18.988
18.253
17.586
17.509
17.290
16.948
16.364
Current Data Parameters
NAME RK2
EXPNO 2
PROCNO 1
F2 - Acquisition Parameters
Date_ 20140206
Time 2.29
INSTRUM spect
PROBHD 5 mm PABBO BB-
PULPROG zgpg30
TD 65536
SOLVENT Pyr
NS 5000
DS 2
SWH 31250.000 Hz
FIDRES 0.476837 Hz
AQ 1.0486259 sec
RG 203
DW 16.000 usec
DE 6.50 usec
TE 300.6 K
D1 2.00000000 sec
D11 0.03000000 sec
======== CHANNEL f1 ========
NUC1 13C
P1 14.43 usec
PLW1 114.01999664 W
SFO1 125.7628195 MHz
======== CHANNEL f2 ========
CPDPRG2 waltz16
NUC2 1H
PCPD2 80.00 usec
PLW2 19.72400093 W
PLW12 0.41828001 W
PLW13 0.26769999 W
SFO2 500.1020004 MHz
F2 - Processing parameters
SI 32768
SF 125.7501582 MHz
WDW EM
SSB 0
LB 1.00 Hz
GB 0
PC 1.40
200
180
160
140
120
100
80
60
40
20
ppm

**Figure S5**

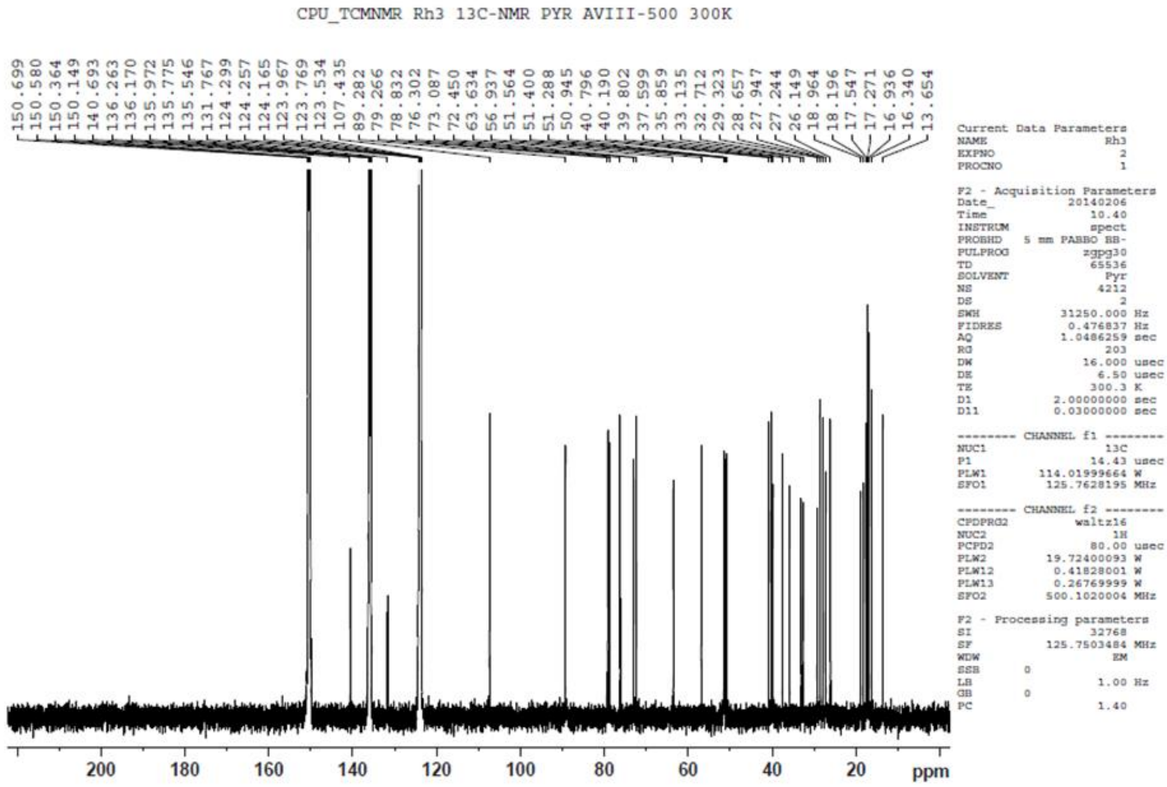

CPU_TCMNMR Rh3 13C-NMR PYR AVIII-500 300K
150.699
150.580
150.364
150.149
140.693
136.263
136.170
135.972
135.775
135.546
131.767
124.299
124.257
124.165
123.967
123.769
123.534
107.435
89.282
79.266
78.832
76.302
73.087
72.450
63.634
56.937
51.564
51.400
51.288
50.945
40.796
40.190
39.802
37.599
35.859
33.135
32.712
29.323
28.657
27.947
27.244
26.149
18.964
18.196
17.547
17.271
16.936
16.340
13.654
Current Data Parameters
NAME Rh3
EXPNO 2
PROCNO 1
F2 - Acquisition Parameters
Date_ 20140206
Time 10.40
INSTRUM spect
PROBHD 5 mm PABBO BB-
PULPROG zgpg30
TD 65536
SOLVENT Pyr
NS 4212
DS 2
SWH 31250.000 Hz
FIDRES 0.476837 Hz
AQ 1.0486259 sec
RG 203
DW 16.000 usec
DE 6.50 usec
TE 300.3 K
D1 2.00000000 sec
D11 0.03000000 sec
======== CHANNEL f1 ========
NUC1 13C
P1 14.43 usec
PLW1 114.01999664 W
SFO1 125.7628195 MHz
======== CHANNEL f2 ========
CPDPRG2 waltz16
NUC2 1H
PCPD2 80.00 usec
PLW2 19.72400093 W
PLW12 0.41828001 W
PLW13 0.26769999 W
SFO2 500.1020004 MHz
F2 - Processing parameters
SI 32768
SF 125.7503484 MHz
WDW EM
SSB 0
LB 1.00 Hz
GB 0
PC 1.40
200
180
160
140
120
100
80
60
40
20
ppm


**Figure S6**

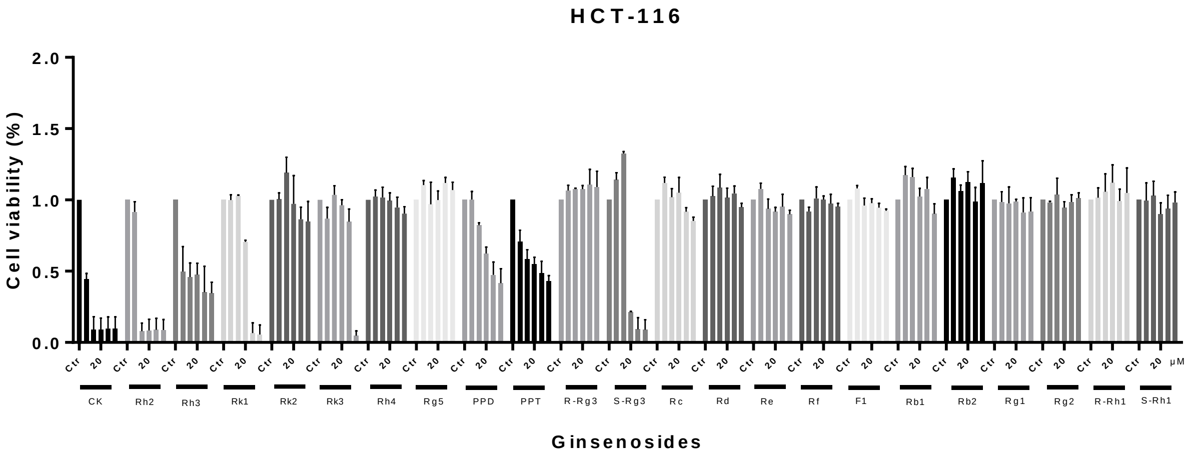
HCT-116
Cell viability (%)
2.0
1.5
1.0
0.5
0.0
Ctr
20
μM
CK
Rh2
Rh3
Rk1
Rk2
Rk3
Rh4
Rg5
PPD
PPT
R-Rg3
S-Rg3
Rc
Rd
Re
Rf
F1
Rb1
Rb2
Rg1
Rg2
R-Rh1
S-Rh1
Ginsenosides

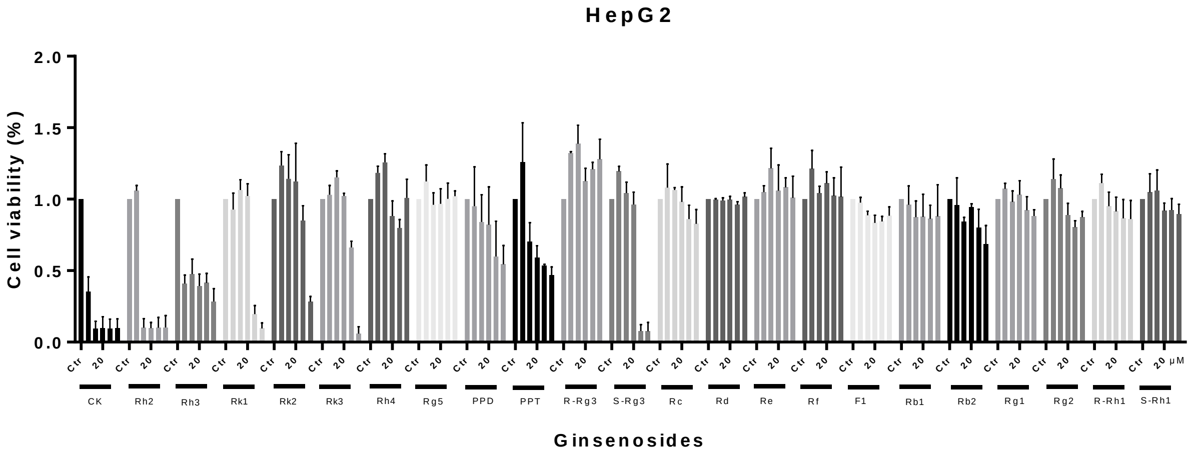
HepG2
Cell viability (%)
2.0
1.5
1.0
0.5
0.0
Ctr
20
μM
CK
Rh2
Rh3
Rk1
Rk2
Rk3
Rh4
Rg5
PPD
PPT
R-Rg3
S-Rg3
Rc
Rd
Re
Rf
F1
Rb1
Rb2
Rg1
Rg2
R-Rh1
S-Rh1
Ginsenosides

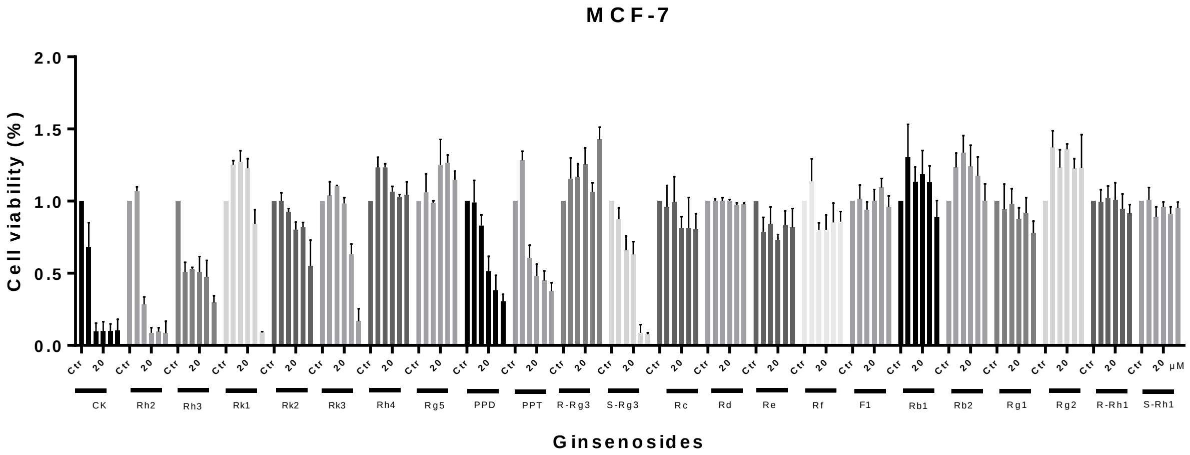
MCF-7
Cell viability (%)
2.0
1.5
1.0
0.5
0.0
Ctr
20
μM
CK
Rh2
Rh3
Rk1
Rk2
Rk3
Rh4
Rg5
PPD
PPT
R-Rg3
S-Rg3
Rc
Rd
Re
Rf
F1
Rb1
Rb2
Rg1
Rg2
R-Rh1
S-Rh1
Ginsenosides

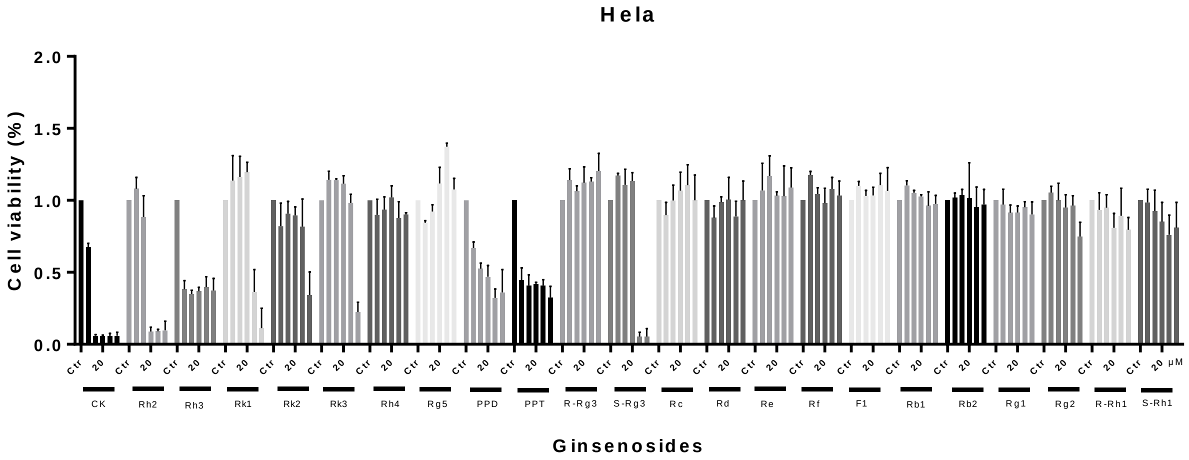
Hela
Cell viability (%)
2.0
1.5
1.0
0.5
0.0
Ctr
20
μM
CK
Rh2
Rh3
Rk1
Rk2
Rk3
Rh4
Rg5
PPD
PPT
R-Rg3
S-Rg3
Rc
Rd
Re
Rf
F1
Rb1
Rb2
Rg1
Rg2
R-Rh1
S-Rh1
Ginsenosides

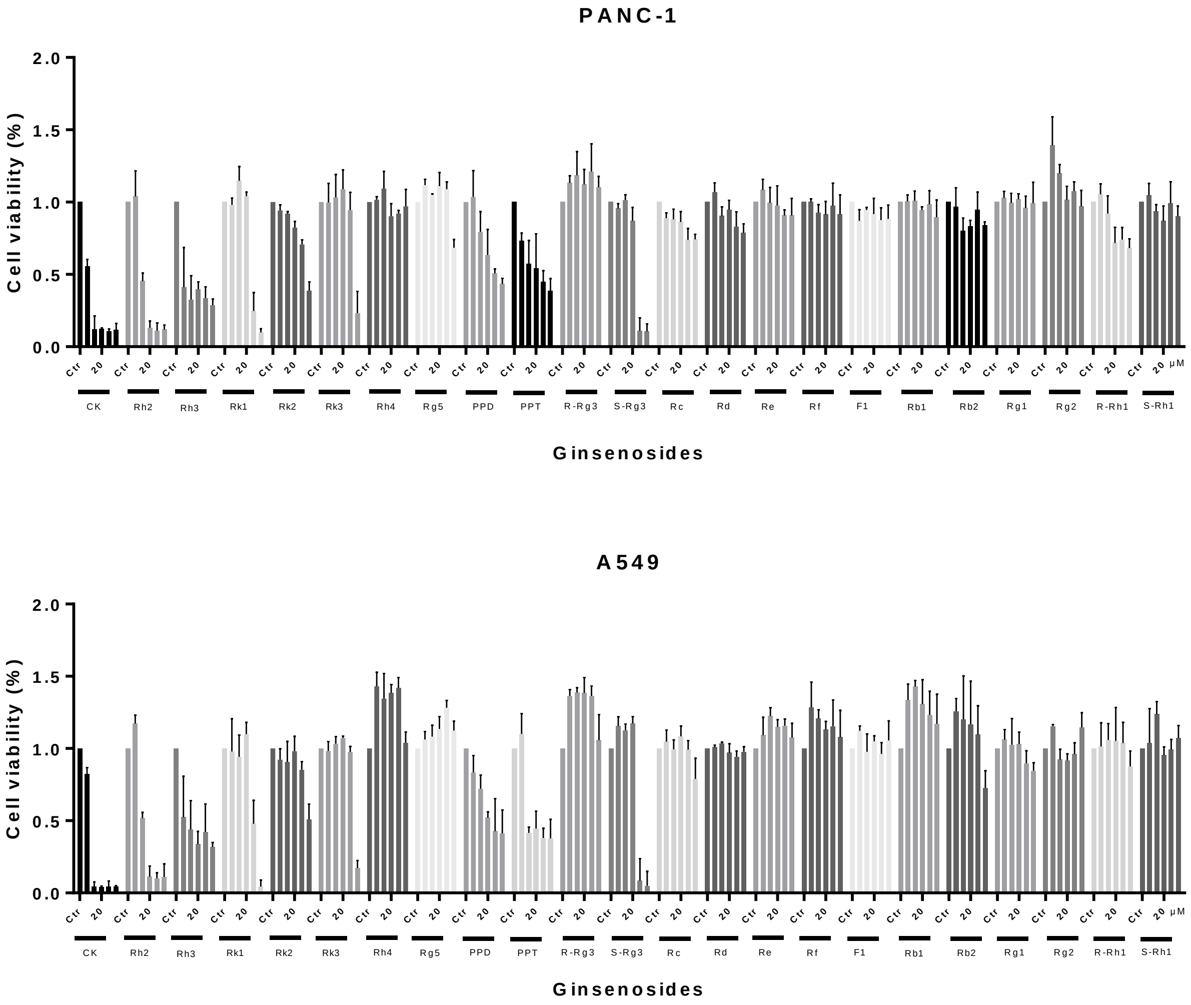
PANC-1
Cell viability (%)
2.0
1.5
1.0
0.5
0.0
Ctr
20
μM
CK
Rh2
Rh3
Rk1
Rk2
Rk3
Rh4
Rg5
PPD
PPT
R-Rg3
S-Rg3
Rc
Rd
Re
Rf
F1
Rb1
Rb2
Rg1
Rg2
R-Rh1
S-Rh1
Ginsenosides
A549
Cell viability (%)
2.0
1.5
1.0
0.5
0.0
Ctr
20
μM
CK
Rh2
Rh3
Rk1
Rk2
Rk3
Rh4
Rg5
PPD
PPT
R-Rg3
S-Rg3
Rc
Rd
Re
Rf
F1
Rb1
Rb2
Rg1
Rg2
R-Rh1
S-Rh1
Ginsenosides

**Figure S7**
